# Effect of frozen storage on the biochemical composition of five commercial freshwater fish species from River Nile, Sudan

**DOI:** 10.1002/fsn3.2340

**Published:** 2021-05-26

**Authors:** Inass A. Malik, Elgasim A. Elgasim, Oladipupo Q. Adiamo, Asmahan Azhari Ali, Isam A. Mohamed Ahmed

**Affiliations:** ^1^ Department of Food Science and Technology Faculty of Agriculture University of Khartoum Khartoum North Sudan; ^2^ Center for Nutrition and Food Sciences Queensland Alliance for Agriculture and Food Innovation University of Queensland Brisbane Qld Australia; ^3^ Department of Food Science and Human Nutrition College of Agriculture and Veterinary Medicine Qassim University Qassim Saudi Arabia; ^4^ Department of Food Science and Nutrition Faculty of Food and Agricultural Sciences King Saud University Riyadh Saudi Arabia

**Keywords:** biochemical composition, freshwater fish species, frozen storage, mineral contents

## Abstract

Postharvest processing and preservation of fish have great influence on fish quality and consumption. Freshwater fish in Sudan are facing problems related to bad handling and improper storage which reduce their quality. This study investigated the changes in the chemical composition, mineral contents, pH and acid value during storage (−18°C) of five commercial fish species (*Bagras bayad*, *Lates niloticus* L., *Mormyrus casahive* L., *Oreochromis nilotica* L., and *Synodrontis schall*) from the River Nile coast of Sudan. The fish species are rich in protein (17.22%–23.60%) but have low fat and ash contents. Frozen storage of the fishes for 45 days reduces their protein contents while the fat and ash contents were increased (*p* ≤ .05). Potassium and iron are the predominant major and trace minerals and their values were increased with storage period. The pH range from 5.74 (*O. niloticus*) to 6.24 (*B. bayad*) while acid value range from 0.02 (*M*. *casahive*) to 0.12 (*L. niloticus*). Both pH and acid values increased with storage period. In conclusion, storage of these fish species for up to 45 days did not adversely affect their nutritional value.

## Graphical abstract

Freshwater fish in Sudan are facing problems related to bad handling and improper storage which reduce their quality. This study investigated the changes in the chemical composition, mineral contents, pH, and acid value during storage (−18°C) of five commercial fish species from the River Nile coast of Sudan. Storage of these fish species for up to 45 days did not adversely affect their nutritional value.

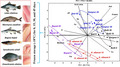

## 1 INTRODUCTION

Generally, fish has been an important source of foods for human since it is among the cheapest source of protein, highly digestible and rich in unsaturated fatty acids. Moreover, it contains high amount of other nutrients such as minerals and essential amino acids for the development of functional and structural proteins (Mahboob et al., 2015). Increase in human population has led to increase in demand for alternative source of proteins and the gap in fish supplies and as human food are expected to be filled by aquaculture industries (Naylor et al., 2000).

The River Nile in Sudan is rich in fish biodiversity with approximately of 128 species belonging to 27 families (Witte et al., 2009). In spite of high fish potentialities in Sudan, yet consumption of fish meat is still quite low compared to that of red meat and this may probably be due to poor postharvest processing and preservation. Freshwater fish products are known to easily deteriorate during post mortem storage and processing as a result of autolytic degradation, microbiological spoilage, and lipid oxidation (Akter et al., 2012; Subramanian, 2007). Also, freshwater fish and its products in Sudan are facing problems related to bad handling and improper storage which reduce their quality. Inadequate storage techniques would imply a substantial shortfall in fish availability thereby affecting the animal protein intake of the people and has a large economic impact (Adejonwo et al., 2010).

Freezing storage is an important postmortem method for preservation of fish and it is considered as the best way of preserving the quality of fish if proper care at each step of freezing is done (Foucat et al., 2001). Fish quality also deteriorate to some extend during freezing storage and the extent of deterioration is usually determined by measuring the sensory, chemical, and physical changes (Liu et al., 2009; Obemeata et al., 2011). In order to increase the utilization of commonly consumed fresh water fishes obtained from River Nile in Sudan, changes in quality of the fishes during freezing storage need to be examined. Therefore, the present study evaluated the changes in the chemical composition, pH, acid value, and mineral composition of five commercial fishes; Bulti (*Oreochromis nilotica*), Gargour (*Synodrontis schall*), Khasm elbanat (*Mormyrus casahive*), Ijeel (*Mormyrus casahive*), and Byad (*Bagras bayad*) present in River Nile in Sudan.

## 2 MATERIALS AND METHODS

### 2.1 Collection of samples

Five species of freshwater fish from River Nile commonly consumed in Sudan were used in this study. Families, genera, species as well as local and English names of these fishes are presented in Table 1. Fresh fish samples (4 kg each) of the same freshness, catch period, weight (480–600 g/fish), and approximate length (25–30 cm/fish) were collected randomly from fishermen. The samples were then transported within 2–3 hr in ice boxes (4°C) to the laboratories of Department of Food Science and Technology, Faculty of Agriculture, University of Khartoum. All reagents used were of analytical grade and procured from Fisher Scientific Co., Rochester, New York.

**TABLE 1:** The types of the fish species used in this study

Family	Genus	Species	Local name	English name
Bagridae	Bagrus	*Bagrus bayad*	Bayad	Forskal's catfish
Centropomidae	Lates	*Lates niloticus* (L.)	Ijeel	Nile Perch
Mormyridae	Morymyrus	*Mormyrus casahive* (L.)	Khashm elbanat	Elephant Snout
Cichlidae	Tilapia	*Oreochromis nilotica* (L.)	Bulti	Perch
Mochokidae	Synodontis	*Synodontis schall*	Gargur	Bolch‐Schneider

### 2.2 Sample preparation

Fish samples were thoroughly washed with clean water to remove all contaminants or unwanted particles and dirt, disemboweled and beheaded. The nonedible portions (offal, head, viscera, and scales) were removed and weighed to determine percentage of edible portion in each fish. Fish muscles were separated from the bones and the leans muscles remaining were chopped into pieces (0.25 cm), homogenate thoroughly and divided into four equal portions. Each portion was assigned randomly to one of four storage period (0, 15, 30, and 45 days), packed in polythene bags and frozen in Gyrofreezer set at −18°C. Samples were taken at each storage period, thawed at room temperature (25°C), and then minced before each analysis.

### 2.3 Chemical composition

The chemical compositions (moisture, crude protein, fat, and ash) of the fresh fishes were determined according to AOAC (2005) method. The moisture content was determined by oven drying method (Method No. 934.01) at 105°C (AOAC, 2005). The crude protein content was determined using Kjeldahl method (Method No. 978.04) and 6.25 was used as conversion factor (AOAC, 2005). The crude fat content was determined using Soxhlet method (Method No. 930.09) and petroleum ether as extracting solvent (AOAC, 2005). The ash content was measured using incineration method (Method No. 930.05) at 550°C (AOAC, 2005). The results were expressed as percentage of wet weight basis.

### 2.4 pH determination

Exactly, 50 ml of distilled water was added to 10 g of fresh minced fish samples. The mixture was stirred well for about 5 min, and contents were then allowed to settle. The pH of the mixture was recorded using HI 255 combined pH meter.

### 2.5 Determination of acid value

Acid value was analyzed according to official method Cd 3a‐63 (AOCS, 2017). One ml of oil or fat extracted from fish samples was transferred into a glass vial and dissolved in mixture of 100 ml of ethanol and diethyl ether (1:1; v/v), heated gently and titrated with shaking against 0.1 M KOH in ethanol, accurately standardized with 0.1 M HCl, using 1% phenolphthalein in 95% C_2_H_5_OH as indicator (5 drops). The end point was recorded when a faint pink color persists for ten seconds. The results were expressed as mg KOH/g. The equation below was used to calculate the acid value.

$$\text{Acid value}=\frac{(A‐B)\times N\times 56.1}{W}$$

where: *A*; vol (mL) of standard KOH used in titrating the sample, *B*; vol (mL) of standard KOH used in titrating the blank, *N*; normality of standard KOH, 56.1; molecular weight of KOH, and *W*; weight of the sample.

### 2.6 Determination of mineral contents

The minerals [sodium (Na), potassium (K), iron (Fe), zinc (Zn), copper (Cu), and calcium (Ca)] were extracted using dry ashing method no. 942.05 (AOAC, 2005). Samples were ashed at 550°C to a constant weight, dissolved with distilled water and few drops of concentrated HCl were added. Sodium and potassium elements were determined using a flame photometer (Model PFP7). Calcium, Fe, Zn, and Cu were calculated by Atomic Absorption Spectrophotometer (Pekin‐Elmar, 3110, USA).

### 2.7 Statistical analysis

Data were analyzed using the Software of the Statistical Analysis System (SAS). Experiments were carried out in triplicates. The data were assigned in Completely Randomized Design (CDR) and subjected to one‐way analysis of variance (ANOVA). Mean separation was done using DMRT tests and significance level was accepted at the probability level of (*p* ≤ .05).

## 3 RESULTS AND DISCUSSION

### 3.1 Effect of species and storage on chemical composition of fish fillets

The chemical composition of fishes could greatly influence the postharvest processes and storage and also assist in determining the suitability of different species to specific processing and storage techniques (Optstvedt, 2000). As shown in Table 2, moisture content of all studied species was gradually decreased as the storage time progressed. All samples showed the highest moisture contents before freezing (zero time), while the lowest moisture contents were observed at the end of storage period (45 days). Similar trends of reduction were reported by Arannilewa et al. (2005) who stored Tilapia fish fillets for up to 60 days at −18°C. Regardless of storage period, the highest (79.10%) moisture content was observed in Bayad at day zero while the lowest (71.82%) value was found in Khasm elbanat at day 45. Regarding Gargour, Ijeel and Khasm elbanat, the moisture content was significantly (*p* ≤ .05) dropped after 15 days of storage, however, that of Bulti and Bayad remained unchanged during 15 days of storage. Further reduction (*p* ≤ .05) in moisture content occurred in Gargour and Ijeel at the 30th day of freezing and then remained constant till the end of the storage period. Similarly, constant moisture content was observed in tail meat of the giant river prawn stored in ice without direct contact for 14 days (Kirschnik et al., 2006). The decline in moisture content during the earlier stages of storage might be due to sublimation of surface water of fish in the freezer (Gandotra et al., 2012). However, moisture losses in the later stage of freezing may be due to myofibrillar distortion resulting in poor water denaturation ability in the muscles of fish fillets (Gandotra et al., 2012). In addition, water loss by evaporation is greater during the precooling period, since the mass transfer rate is reduced when the product freeze. The high moisture content of the fish fillet would increase the deterioration level of fish when kept for long time due to the increased activity of the microorganisms associated with the high moisture content (Love, 1980; Oluwaniyi & Dosumu, 2009). Generally, storage at −18°C for 45 days reduced the moisture content of the investigated samples with a reduction rate of 0.77% (Bulti) to 1.95% (Gargour). Storage time and temperature are the major factors implicated in the loss of quality and shelf life of fish (Rahman, 2007) which result in hard, dry and fibrous fish products, hence reduced eating quality. Moreover, liquid losses may also directly result in economic losses (Obuz and Dikeman, 2003).

**TABLE 2:** Effect of species and storage period on approximate composition (%, wet weight basis) of five commercial fish species from Nile River, Sudan

Fish species	Storage period (days)				
	0	15	30	45	Species average
Moisture					
Bulti (*O. niloticus*)	74.30^d^ ± 0.17	74.05^de^ ± 0.04	73.76^e^ ± 0.05	73.73^e^ ± 0.02	74.00^B^ ± 0.17
Gargour (*S*. *schall*)	73.89^e^ ± 0.07	73.07^f^ ± 0.02	72.56^g^ ± 0.02	72.47^g^ ± 0.02	73.00^D^ ± 0.05
Khasm elbanat (*M. casahive*)	72.96^f^ ± 0.28	72.41^g^ ± 0.36	72.31^g^ ± 0.06	71.82^h^ ± 0.28	72.40^E^ ± 0.30
Ijeel (*L*. *niloticus*)	74.20^d^ ± 0.01	73.84^e^ ± 0.28	72.79^fg^ ± 0.59	72.82^fg^ ± 0.10	73.50^C^ ± 0.07
Byad (*B. bayad*)	79.10^a^ ± 0.04	78.86^ab^ ± 0.01	78.60^b^ ± 0.07	78.16^c^ ± 0.29	78.70^A^ ± 0.04
Storage average	74.91^A^ ± 1.59	74.45^B^ ± 2.02	74.00^C^ ± 2.32	73.80^D^ ± 2.51	
Protein					
Bulti (*O. niloticus*)	22.90^bc^ ± 0.05	22.80^bc^ ± 0.03	21.64^d^ ± 0.63	20.87^e^ ± 0.44	22.02^C^ ± 0.05
Gargour (*S*. *schall*)	23.60^a^ ± 0.02	22.80^bc^ ± 0.30	22.43^c^ ± 0.10	22.40^c^ ± 0.08	22.80^A^ ± 0.02
Khasm elbanat (*M. casahive*)	19.94^f^ ± 0.20	18.31^g^ ± 0.12	17.10^hi^ ± 0.3	16.69^i^ ± 0.08	18.01^D^ ± 0.20
Ijeel (*L*. *niloticus*)	23.03^b^ ± 0.20	22.85^bc^ ± 0.22	22.66^bc^ ± 0.21	22.20^c^ ± 0.13	22.70^B^ ± 0.30
Byad (*B. bayad*)	17.22^h^ ± 0.01	17.11^hi^ ± 0.01	17.00^hi^ ± 0.02	16.62^i^ ± 0.03	17.00^E^ ± 0.01
Storage average	21.32^A^ ± 2.30	20.80^B^ ± 2.45	20.16^C^ ± 2.60	19.75^D^ ± 2.70	
Fat					
Bulti (*O. niloticus*)	1.44^k^ ± 0.04	1.62^k^ ± 0.02	1.98^j^ ± 0.06	2.18^i^ ± 0.16	1.81^E^ ± 0.04
Gargour (*S*. *schall*)	1.53^k^ ± 0.01	1.93^j^ ± 0.04	2.53^h^ ± 0.01	2.55^h^ ± 0.02	2.13^D^ ± 0.01
Khasm elbanat (*M. casahive*)	6.06^d^ ± 0.02	8.07^c^ ± 0.01	9.03^b^ ± 0.03	10.13^a^ ± 0.3	8.32^A^ ± 0.02
Ijeel (*L*. *niloticus*)	0.98^l^ ± 0.03	2.40^hi^ ± 0.10	3.15^fg^ ± 0.08	3.32^f^ ± 0.05	2.46^C^ ± 0.02
Byad (*B. bayad*)	2.23^i^ ± 0.02	3.04^g^ ± 0.02	3.19^fg^ ± 0.01	4.07^e^ ± 0.03	3.13^B^ ± 0.02
Storage average	2.45^D^ ± 2.80	3.41^C^ ± 2.75	3.98^B^ ± 2.71	4.45^A^ ± 2.67	
Ash					
Bulti (*O. niloticus*)	1.42^bc^ ± 0.04	1.51^ab^ ± 0.03	1.50^ab^ ± 0.01	1.55^a^ ± 0.02	1.5^A^ ± 0.04
Gargour (*S*. *schall*)	1.42^bc^ ± 0.01	1.47^b^ ± 0.01	1.48^ab^ ± 0.00	1.56^ab^ ± 0.03	1.48^AB^ ± 0.05
Khasm elbanat (*M. casahive*)	1.08^e^ ± 0.00	1.10^e^ ± 0.04	1.17^de^ ± 0.00	1.20^d^ ± 0.02	1.14^C^ ± 0.02
Ijeel (*L*. *niloticus*)	1.38^c^ ± 0.05	1.47^b^ ± 0.16	1.52^ab^ ± 0.04	1.52^ab^ ± 0.05	1.47^B^ ± 0.01
Byad (*B. bayad*)	1.03^e^ ± 0.01	1.05^e^ ± 0.03	1.07^e^ ± 0.05	1.10^e^ ± 0.10	1.06^D^ ± 0.01
Storage average	1.27^D^ ± 0.17	1.32^C^ ± 0.19	1.35^B^ ± 0.20	1.39^A^ ± 0.20	

Note

Means (*n* = 3) not sharing a common letter in columns and rows are significantly different at *p* ≤ .05.

Nearly all frozen fish fillet samples stored for 45 days showed significantly (*p* ≤ .05) lower protein contents compared to fresh samples (zero time). It is clear that protein content of fillet samples changed significantly (*p* ≤ .05) at some stages of storage (from 23.60%, for unfrozen Gargour to 16.62%, for Bayad in the 45th day of storage), with a reduction rate of 3.53%–17.75% for all studied species. Similar trend of reduction was observed in Iranian fish that was frozen for 60 days (Aberoumand, 2013). With respect to Khasm elbanat and Gargour a significant (*p* ≤ .05) decrease in protein content was first detected at15th day of storage. Whereas, for both Bulti and Ijeel a significant (*p* ≤ .05) reduction in protein was first detected at the 30th day of storage. Interestingly, both Ijeel and Bayad resisted changes in protein content for about 45 days of storage. Likewise, several researchers observed that protein decreased during frozen storage and this reduction in protein was ascribed to denaturation (Aberoumand, 2013; Arekemase et al., 2010; Ekpenyong & Ibok, 2012; Saliu, 2008) and to changes in the proportion of chemical composition and protein breakdown (Kjærsgård et al., 2006). Also, there may be leaching of nitrogen during thawing which in turn causes a reduction in the protein content (Akter et al., 2012; Arannilewa et al., 2005). Furthermore, fish undergoes rapid protein degradation due to the action of endogenous and bacterial enzymes after death of fishes (Saeed & Howell, 2002). Deterioration of fish quality due to protein denaturation can therefore be checked by using as low temperature as possible, preferably at −18°C. Storage temperature and time have great impact on the degree of protein denaturation (Herrero et al., 2005). The shelf life of fish products, therefore, is markedly extended when products are stored at low temperatures (Herrero et al., 2005). Other possible reasons for decrease in protein content might be the loss of ammonia (NH_3_), volatile amines, conversion of nitrogen to other nonprotein nitrogen molecules, amino acid destruction, and formation of protein–fat complexes that results in decrease in protein content (Saeed & Howell, 2002). Present study shows that storage can cause decrease in the protein content of freshwater fish from River Nile (Sudan). Thus, it is better to consume the fishes before 45 day of storage or to be involved in a technological process after freezing at −18°C for 45 days.

The fat contents of fillet samples of all examined species, excluding Bulti, were significantly (*p* ≤ .05) increased throughout the storage period compared to fresh samples (Table 2). Although, fat content of Bulti was not changed at 15th day of storage, however, it increased (*p* ≤ .05) significantly as the storage period progressed. Highest fat content was found in all the species at the end of the storage period. Similar increases in fat content during storage have been reported in Tilapia fish fillets (Emire & Gebremariam, 2010) and sea bass fillets (Özyurt et al., 2005). Although the fat content was increased in all fishes as the storage period elevated, but the trend of increasing differed significantly (*p* ≤ .05) between fish species. The resultant increased fat content can be ascribed to the fact that there is an inverse relationship (*r* = −0.99) between the moisture and lipid contents of fish flesh (Begum et al., 2012; Inhamuns & Bueno Franco, 2001; Oliveira et al., 2003).

The highest ash contents for all investigated fish species were perceived in the 45th day of the storage, while the lowest ash values were recorded in the initial day of storage (Table 2). However, the differences were not significant except in Bulti and Ijeel at the end of storage period as compared to day 0. Similarly, higher ash percentage was found in Tilapia fish after 240 days of storage at −18°C as compared to fresh sample. Conversely, Okeyo et al. (2009) who studied *L. niloticus* fish found that ash content was not significantly (*p* ≥ .05) related to storage time. Loss of water in any food substances produce an uneven increase in the percentage of other nutrients (Castrillon et al., 2008), therefore, the consequential increase in ash content during freezing might be due to the decrease in moisture and protein contents of the studied fish samples.

### 3.2 Effect of species and storage on mineral composition of fish fillets

The five fish species showed different mineral concentrations throughout the different storage intervals (Table 3). Fish absorb minerals not only from their diets but also from the surrounding water via their gills and skin (Nurnadia et al., 2013). As shown in Table 3, both Bulti and Khasm elbanat showed no significant variations in their Na, K, Ca, and Fe contents through the entire period of storage. Zinc content of the fresh Khasm elbanat (1.42 mg/100 g) was not affected by freezing up to the 30th day of storage, but significantly (*p* ≤ .05) decreased to 0.56 mg/100 g at the 45th day. Moreover, the copper contents of the Khasm elbanat and Bulti were significantly (*p* ≤ .05) declined at the 15th day of storage, with lowest value obtained at the end of the storage period. Minerals contents of Gargour and Ijeel showed no significant changes along the duration of the study except Zn and Cu contents. Storage of Gargour and Ijeel species significantly (*p* ≤ .05) reduced their Cu contents after 15 days of storage and further reduction was observed only on Gargour at the end of the storage period. However, there was stability in K and Zn contents in Ijeel and Ca content in Gargour, during the first 4 weeks of storage. At the end of the storage period, Zn of Ijeel and Ca of Gargour was significantly (*p* ≤ .05) declined, while K of Ijeel was significantly (*p* ≤ .05) elevated. As regards to Bayad specie, storage did not cause any significant changes on the Ca and Zn contents, but its K and Fe contents were increased significantly (*p* ≤ .05) after 30 days of storage. Also, significantly (*p* ≤ .05) higher Na content was found in Bayad after 45 days of storage while the Cu content decreased at the end of storage period as compared to fresh sample. The variation in minerals contents of fish species during storage condition could be attributed to differences in concentration of the mineral ions in the fish fleshes, feeding behavior, environment, ecosystem, and migration. In addition, minerals availabilities in the water body, chemical form of the element and the ability of the fish to absorb these inorganic elements from their diet could also affect the mineral contents of freshwater fishes during frozen storage. Wolfe and Bryant (2001) attributed the losses of mineral during slow freezing to the separation of water from colloidal solution or plasma and its conversion to pure ice. This phenomenon creates osmotic imbalance, as remaining solution becomes highly concentrated with dissolved salts. Consequently, intracellular water starts flowing out along with nutrients, which drains out during thawing process. Muhammad and Ajiboye (2010) attributed the considerable variations among minerals during storage to the leaching of the minerals during the dewatering and washing stages. Mahmoud Sharaf (2013) found no clear relationship between the mineral contents and the different freezing periods of Tilapia muscles at −18°C. Similarly, Arannilewa et al. (2005) observed slight changes in minerals and attributed that to the drip loss and the dehydration associated with frozen storage. Generally, the impact of storage on the mineral contents of freshwater fishes was differed depending on the types of species and studied minerals as well as storage period.

**TABLE 3:** Effect of specie and storage on the mineral contents (mg/100 g) of fresh water fishes from River Nile, Sudan

Fish species	Storage period (days)				
	0	15	30	45	Species average
K					
Bulti (*O. niloticus*)	333.30^c^ ± 0.04	408.30^bc^ ± 0.02	368.30^c^ ± 0.06	343.30^c^ ± 0.16	363. 30^C^ ± 0.04
Gargour (*S*. *schall*)	373.30^c^ ± 0.02	380.00^c^ ± 0.04	366.70^c^ ± 0.01	356.30^c^ ± 0.02	369.20^C^ ± 0.02
Khasm elbanat (*M. casahive*)	401.70^bc^ ± 0.00	416.70^bc^ ± 0.54	493.30^ab^ ± 0.62	513.30^ab^ ± 0.40	456.20^B^ ± 0.00
Ijeel (*L*. *niloticus*)	428.30^bc^ ± 0.86	480.00^ab^ ± 0.52	456.70^b^ ± 0.12	548.30^a^ ± 0.70	478.30^A^ ± 0.90
Byad (*B. bayad*)	368.30^c^ ± 0.66	378.30^c^ ± 0.02	546.70 ^a^ ± 0.01	508.30^ab^ ± 0.03	450.40^B^ ± 0.42
Storage average	381.00^C^ ± 64.60	412.70^B^ ± 61.80	446.30^A^ ± 69.10	454.00^A^ ± 70.00	
Na					
Bulti (*O. niloticus*)	78.30^d^ ± 15.2	85.00^d^ ± 13.20	101.70^cd^ ± 3.00	95.00^cd^ ± 5.00	90.00^D^ ± 15.20
Gargour (*S*. *schall*)	90.00^d^ ± 5.00	118.33 cd ± 3.00	81.70^d^ ± 16.00	83.30^d^ ± 14.40	93.30^D^ ± 5.00
Khasm elbanat (*M. casahive*)	96.60^cd^ ± 3.00	128.33^cd^ ± 3.00	120.00^cd^ ± 5.00	113.30^cd^ ± 12.50	114.50^C^ ± 3.00
Ijeel (*L*. *niloticus*)	206.60^a^ ± 3.00	221.70^a^ ± 10.00	226.60^a^ ± 15.20	226.70^a^ ± 7.630	220.40^A^ ± 10.40
Byad (*B. bayad*)	98.30^cd^ ± 3.00	113.33^cd^ ± 13.20	160.00^bc^ ± 5.00	166.70^b^ ± 17.50	134.50^B^ ± 3.00
Storage average	114.00^B^ ± 54.40	133.30^A^ ± 53.00	138.00^A^ ± 52.20	137.00^A^ ± 52.18	
Ca					
Bulti (*O. niloticus*)	6.05^c^ ± 0.80	6.25^c^ ± 0.00	4.96^c^ ± 0.05	6.15^c^ ± 0.06	5.85^E^ ± 0.77
Gargour (*S*. *schall*)	10.40^bc^ ± 0.40	12.16^ab^ ± 1.01	11.9^ab^ ± 0.74	6.44^c^ ± 0.14	10.24^C^ ± 0.40
Khasm elbanat (*M. casahive*)	6.87^c^ ± 1.22	7.71^c^ ± 0.17	7.16^c^ ± 0.22	9.47^bc^ ± 0.29	7.80^D^ ± 1.20
Ijeel (*L*. *niloticus*)	13.46^ab^ ± 1.06	12.82^ab^ ± 0.86	12.44^ab^ ± 0.33	14.13^a^ ± 0.07	13.21^A^ ± 1.00
Byad (*B. bayad*)	13.2^ab^ ± 0.50	11.2^ab^ ± 0.07	10.97^b^ ± 2.90	9.74^bc^ ± 0.09	11.27^B^ ± 0.50
Storage average	9.99^A^ ± 3.17	10.02^A^ ± 3.07	9.50^B^ ± 3.00	9.19^B^ ± 2.70	
Zn					
Bulti (*O. niloticus*)	1.57^bc^ ± 0.06	1.75^bc^ ± 0.22	1.75^bc^ ± 0.04	1.51^bc^ ± 0.32	1.64^A^ ± 0.28
Gargour (*S*. *schall*)	2.59^a^ ± 0.19	1.26^bc^ ± 0.02	1.58^bc^ ± 0.25	0.59^d^ ± 0.17	1.50^B^ ± 0.17
Khasm elbanat (*M. casahive*)	1.42^bc^ ± 0.10	1.63^bc^ ± 0.11	1.28^bc^ ± 0.06	0.56^d^ ± 0.45	1.22^C^ ± 0.17
Ijeel (*L*. *niloticus*)	1.31^bc^ ± 0.00	1.58^bc^ ± 0.19	1.78 ^b^ ± 0.14	0.53^d^ ± 0.05	1.30^C^ ± 0.00
Byad (*B. bayad*)	0.98^cd^ ± 0.05	1.25^c^ ± 0.24	0.75^cd^ ± 0.00	0.50^d^ ± 0.00	0.87^D^ ± 0.03
Storage average	1.57^A^ ± 1.72	1.49^B^ ± 1.65	1.42^B^ ± 1.88	0.74^C^ ± 1.97	
Cu					
Bulti (*O. niloticus*)	0.62^a^ ± 0.28	0.42^bc^ ± 0.50	0.39^c^ ± 0.05	0.10^ef^ ± 0.20	0.38^B^ ± 0.28
Gargour (*S*. *schall*)	0.46^bc^ ± 0.17	0.28^d^ ± 0.35	0.50^b^ ± 0.41	0.18^e^ ± 0.24	0.36^C^ ± 0.18
Khasm elbanat (*M. casahive*)	0.64^a^ ± 0.17	0.39^c^ ± 0.18	0.50^b^ ± 0.36	0.06^f^ ± 0.02	0.40^B^ ± 0.17
Ijeel (*L*. *niloticus*)	0.63^a^ ± 0.33	0.35^cd^ ± 0.28	0.47^bc^ ± 0.34	0.35^cd^ ± 0.11	0.45^A^ ± 0.33
Byad (*B. bayad*)	0.58^ab^ ± 0.13	0.41^bc^ ± 0.16	0.07^f^ ± 0.02	0.04^f^ ± 0.05	0.27^D^ ± 0.16
Storage average	0.58^A^ ± 0.52	0.39^B^ ± 0.52	0.37^B^ ± 0.53	0.14^C^ ± 0.55	
Fe					
Bulti (*O. niloticus*)	0.70^d^ ± 0.05	0.81^d^ ± 0.06	0.69^d^ ± 0.03	0.94^cd^ ± 0.04	0.77^C^ ± 0.05
Gargour (*S*. *schall*)	1.19^cd^ ± 0.20	1.30^cd^ ± 0.11	1.27^cd^ ± 0.02	1.47^c^ ± 0.13	1.31^B^ ± 0.20
Khasm elbanat (*M. casahive*)	0.41^d^ ± 0.02	0.39^d^ ± 0.02	0.72^d^ ± 0.06	0.79^d^ ± 0.06	0.58^D^ ± 0.02
Ijeel (*L*. *niloticus*)	0.59^d^ ± 0.07	0.53^d^ ± 0.05	1.18 cd ± 0.01	0.63^d^ ± 0.08	0.73^C^ ± 0.07
Byad (*B. bayad*)	3.45^b^ ± 0.27	3.02^b^ ± 0.41	4.51^a^ ± 0.44	4.4^a^ ± 0.36	3.87^A^ ± 0.27
Storage average	1.26^B^ ± 0.70	1.21^B^ ± 0.84	1.68^A^ ± 1.16	1.66^A^ ± 1.36	

Note

Means (*n* = 3) not sharing a common letter in columns and rows are significantly different at *p* ≤ .05.

### 3.3 Effect of species and storage on pH and acid values of fish fillets

The pH and acid value follows the same trend throughout the storage period for all samples (Table 4). The pH values at zero day of all examined samples were significantly (*p* ≤ .05) increased after 15 days of storage. Subsequently, a significant (*p* ≤ .05) increase in pH occurred at the 30th day of storage of all species. Further significant (*p* ≤ .05) rise in pH took place at the end of the storage period in all species except that of Bayad, which remained constant during the last 2 weeks of storage. Generally, maximum pH values (6.55–7.40) were observed at the end of the storage period in all samples. Present results were in a good agreement with the finding of other authors who studied freshwater fish such as the rainbow trout (Chytiri et al., 2004; Rodríguez et al., 2012), however, slightly different from the values reported by Arannilewa et al. (2005) for the frozen Tilapia fish. However, pH results for Bulti were lower than the finding of Obemeata et al. (2011) who stored *T. guineensis* for 4 weeks at −18°C and reported increased pH from 6.81 to 6.85, which might be due to specie difference. In addition, the variations in the pH between these studies perhaps due to the differences in the catching season, geographical location, fish size, and water composition. The increase in pH values during frozen storage may be due to the accumulation of alkaline compounds, such as ammonia, mainly derived from microbial actions (Liu et al., 2013).

**TABLE 4:** Effect of species and storage period on pH and acidity of Nile fish

Fish species	Storage period (days)				
	0	15	30	45	Species average
pH					
Bulti (*O. niloticus*)	5.74^g^ ± 0.00	6.14^f^ ± 0.02	6.32^e^ ± 0.01	6.55^d^ ± 0.03	6.19^D^ ± 0.05
Gargour (*S*. *schall*)	6.49^d^ ± 0.03	6.91^c^ ± 0.02	7.07^b^ ± 0.01	7.40^a^ ± 0.02	6.97^A^ ± 0.03
Khasm elbanat (*M. casahive*)	5.81^g^ ± 0.03	6.05^f^ ± 0.09	6.47^de^ ± 0.03	6.78^c^ ± 0.1	6.28^C^ ± 0.03
Ijeel (*L*. *niloticus*)	5.79^g^ ± 0.02	6.15^f^ ± 0.06	6.32^e^ ± 0.03	6.83^c^ ± 0.03	6.27^C^ ± 0.1
Byad (*B. bayad*)	6.24^ef^ ± 0.01	6.34^e^ ± 0.04	6.88^c^ ± 0.02	6.91^c^ ± 0.03	6.59^B^ ± 0.05
Storage average	6.01^D^ ± 0.46	6.32^C^ ± 0.42	6.61^B^ ± 0.43	6.89^A^ ± 0.44	
Acid value					
Bulti (*O. niloticus*)	0.04^hi^ ± 0.005	0.22^e^ ± 0.001	0.32^c^ ± 0.003	0.42^a^ ± 0.01	0.25^AB^ ± 0.005
Gargour (*S*. *schall*)	0.05^h^ ± 0.005	0.08 ± ^g^0.008	0.23^e^ ± 0.009	0.44^a^ ± 0.005	0.20^C^ ± 0.006
Khasm elbanat (*M. casahive*)	0.02^i^ ± 0.002	0.03^hi^ ± 0.002	0.05^h^ ± 0.001	0.07^g^ ± 0.001	0.05^D^ ± 0.002
Ijeel (*L*. *niloticus*)	0.12^f^ ± 0.01	0.27^d^ ± 0.01	0.30^c^ ± 0.002	0.34^bc^ ± 0.009	0.3^A^ ± 0.01
Byad (*B. bayad*)	0.04^hi^ ± 0.003	0.24^e^ ± 0.002	0.31^c^ ± 0.003	0.36^b^ ± 0.005	0.24^AB^ ± 0.11
Storage average	0.05^D^ ± 0.14	0.17^C^ ± 0.13	0.24^B^ ± 0.14	0.33^A^ ± 0.14	

Note

Means (*n* = 3) not sharing a common letter in columns and rows are significantly different at *p* ≤ .05.

The acid values obtained from extracted oil of stored fillet samples of Bulti, Gargour, Bayad and Ijeel were significantly (*p* ≤ .05) increased following the initial 15 days of storage to between 0.08 and 0.27 mg KOH/g (Table 4). However, for Khasm elbanat a significant (*p* ≤ .05) increase in acid value was first detected at the 30th day of storage (0.06 mg KOH/g). Consequent significant (*p* ≤ .05) increase in acid value for the remaining species also occurred at the 30th day of storage (0.23–0.32 mg KOH/g). All the species have maximum acid value at the end of the storage period that ranged from 0.07 for Khasm elbanat to 0.44 for Gargour. This trend in increasing acid value agreed with the finding of Haliloǧlu et al. (2004) who noticed progressive rise in acid value during cold storage of mullet fish for 11 days. It is obvious that the increasing rate of acid value during freezing is not the same in all fish species, which is related to the differences in type and quality of fats and chains of PUFA of different species of fish that is changed during freezing (Rasoarahona et al., 2005). Furthermore, the progressively increased acid values during freezing might be due to the hydrolytic reactions of lipids by lipase enzymes originating from microorganisms or biological tissues (Talpur et al., 2010). The acceptable range of acid values is ≤3 mg KOH/g according to recently published by (FAO/WHO, 2013). However, results of the present study were less than the limit, which mean that (the oil of) fish stored at −18°C are suitable for consumption up to 45 days.

### 3.4 Multivariate analysis

**FIGURE 1:**
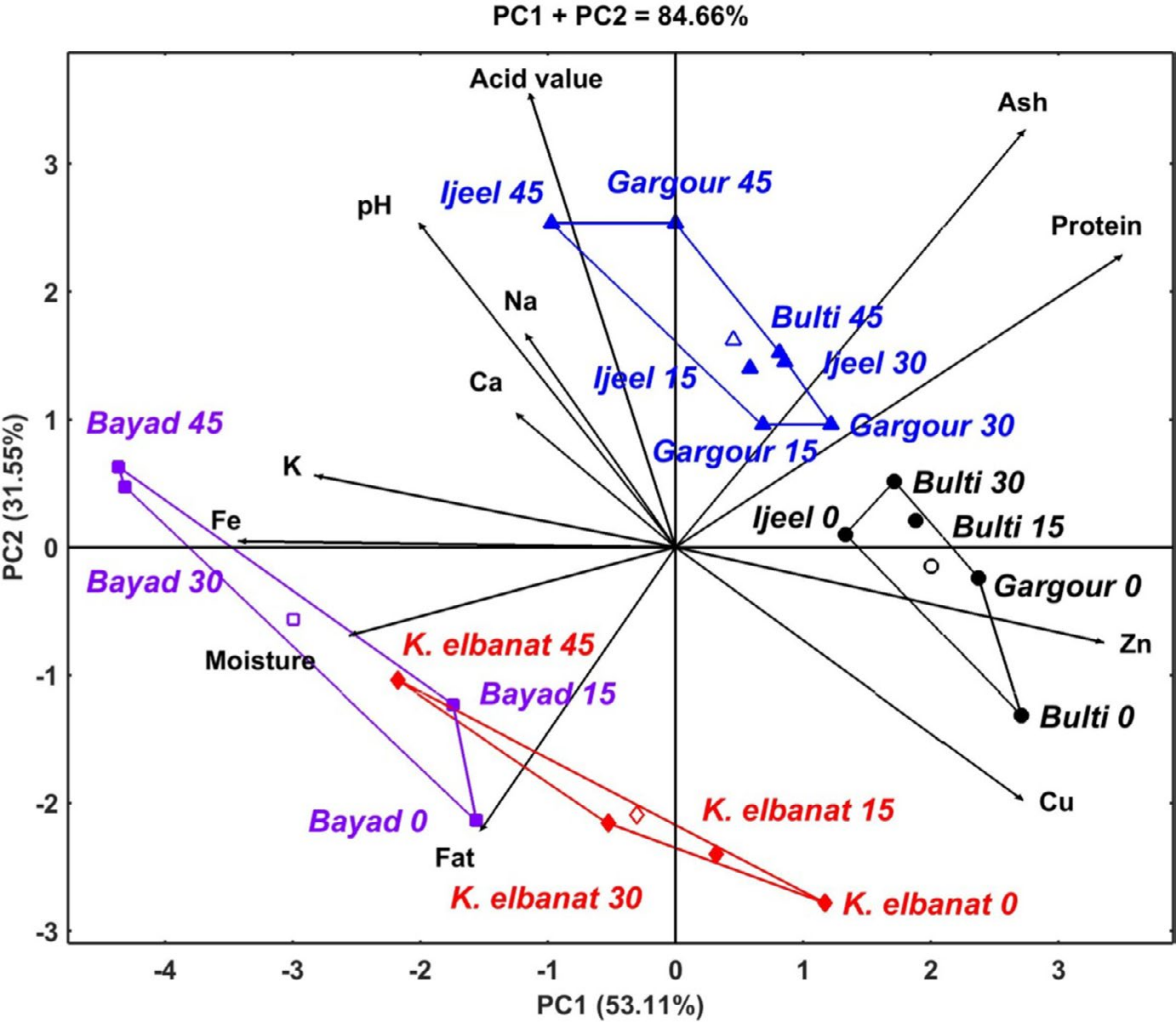
The HJ‐biplot based on principal component analysis for biochemical composition of fleshes of five freshwater fish species from River Nile as affected by frozen storage at −18°C for 0, 15, 30, and 45 days

To profoundly estimate the combined effect of storage period and fish species on the biochemical composition of five freshwater fishes from River Nile, principal component analysis using HJ‐Biplot was conducted. The results clearly showed an excellent contribution of the principle components axes (PC1, 53.11% and PC2, 31.55%) to the total variability (84.66%) of the blotted data (Figure 1). Clustering analysis indicated four groups of fish species and storage times based on their combined impact on the biochemical composition of the fish fleshes. The first group (triangle symbol) composed of Gargour (15, 30, and 45 days), Ijeel (15, 30, and 45 days) and Bulti (at 45 days). This group is characterized by greater ash, protein, acid value, pH, Na, and Ca compared to other species and storage periods. Among this group, Gargour and Ijeel when stored for 45 days developed higher acid value and pH than other fish species suggesting that the fleshes of these species are liable to microbial degradation during storage if stored for period longer than 45 days. The second group (circle symbol) composed of Ijeel (0 day), Gargour (0 day), and Bulti (0, 15, and 45 days). This group is characterized by high Zn and Cu contents with the highest values of these minerals observed in Bulti at day 0 of storage. Regardless of storage time, Bayad and Khasm elbanat formed separate groups, third (squire symbol) and fourth groups (diamond symbol), respectively. Both groups were characterized by high moisture and fat contents, whereas Bayad at 30 and 45 days of storage has high Fe and K contents compared to other species in this group or other groups.

## 4 CONCLUSIONS

In conclusion, the five fish species are good source of protein, potassium, sodium, and iron. Storage of the fishes for up to 45 days greatly reduces their protein contents while their fats, ash, and mineral contents were increased. The increase in pH of the fish species during storage could promote microbial growth, thus, further study should be conducted to investigate the microbiological characteristics of these fishes at these storage conditions.

## CONFLICT OF INTEREST

The authors declare that they have no conflicts of interest.

## ETHICAL APPROVAL

The study did not involve any human or animal testing.

## Data Availability

Data that support the findings of this study will be available from corresponding author upon request.
